# Cholinergic neurotransmitter system: a potential marker for post-stroke cognitive recovery

**DOI:** 10.1093/brain/awac142

**Published:** 2022-04-19

**Authors:** Fatemeh Geranmayeh

**Affiliations:** Clinical Language and Cognition Group, Department of Brain Sciences, Imperial College, London, UK

## Abstract

This scientific commentary refers to ‘Cholinergic and hippocampal systems facilitate cross-domain cognitive recovery after stroke’ by O’Sullivan *et al.* (https://doi.org/10.1093/brain/awac070).

**This scientific commentary refers to ‘Cholinergic and hippocampal systems facilitate cross-domain cognitive recovery after stroke’ by O’Sullivan *et al.* (https://doi.org/10.1093/brain/awac070).**

Stroke is the leading cause of adult disability in Europe, and the number of people living with stroke is expected to increase by a third in the next 30 years. Unsurprisingly, the overall economic burden of stroke is high with an estimated cost of £26 billion per year in the UK alone. Cognitive deficits are a major contributor to post-stroke disability; incident stroke is associated with an acute decline in cognitive functions in addition to an accelerated and persistent cognitive decline over subsequent years. There is therefore a great societal, clinical and scientific need to refine our mechanistic understanding of stroke recovery, a prerequisite to developing more effective therapies.

**Figure 1:**
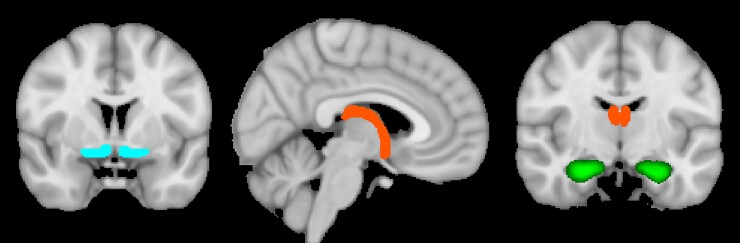
**Schematic illustration of regions discussed in the paper by O'Sullivan and colleagues**. From *left* to *right*: MNI coordinates y = 3; x = 3; y = −10. Blue: Basal forebrain nuclei without subunits from Teipel *et al.*^11^ Green: Hippocampi without subunits from Harvard Oxford subcortical probabilistic atlas provided in FSL and thresholded at 30. Red-yellow: Fornix from XTRACT white matter atlas available in FSL also thresholded at 30. For specific details of regions discussed and their subunits, please see O'Sullivan *et al.*^1^

In this issue of *Brain,* O’Sullivan and colleagues^1^ demonstrate that the fornix, cholinergic basal forebrain nuclei and a set of hippocampal subfields are part of a common infrastructure that facilitates ‘cross domain’ cognitive recovery after stroke. The authors obtained structural MRI measures of grey matter and white matter integrity [using voxel-based morphometry (VBM) and tract-based spatial statistics (TBSS)] in 42 patients with stroke who underwent brain imaging at ∼3 months after the ictus. The imaging measures were related to spontaneous recovery of three cognitive domains, namely long-term, short-term, and working memory, over the subsequent 9 months. Whilst whole brain VBM alone showed no association between grey matter volume and measures of memory recovery, whole brain TBSS suggested an association between the body and column of the fornix and recovery of long term and working memory.

Based on *a priori* knowledge of hippocampal connectivity with the fornix, and cholinergic innervation of distributed brain systems supporting memory, the authors then chose to examine the volume of hippocampal subfields and cholinergic basal forebrain nuclei. Using support vector regression models, the status of the fornix, cholinergic basal forebrain and a set of hippocampal subfields was able to explain a considerable amount of variability in the recovery of both long-term and working memory (62% and 41%, respectively) (Fig. 1). These findings contrast with prior research that posits (not necessarily exclusively) that working memory is supported by a frontoparietal network of brain regions, and are consistent with the view that the cholinergic and hippocampal-fornix system are part of a common infrastructure that supports recovery across multiple cognitive domains.

This work provides significant insight into the mechanisms of spontaneous recovery after stroke and accords well with our understanding of the pathophysiology of Alzheimer’s disease. The role of the cholinergic system in both episodic and working memory is well established. Cholinergic antagonists have clear negative effects on performance of both classical working memory and episodic memory tasks. Greater volume of cholinergic basal forebrain nuclei has been associated with better memory recall in patients with mild cognitive impairment who have fornix atrophy, suggesting that the cholinergic inputs are needed for adaptation to structural compromise of the fornix.^2^ Furthermore, animal work suggests that the cholinergic system is vital for cortical plasticity and motor learning,^3^ in keeping with the notion that the cholinergic system is part of a common machinery for learning and ‘relearning’ after brain injury.

Of course, there are several major questions that remain unanswered by this work. From a systems neuroscience perspective, stroke recovery is likely to be a much more complex affair, mediated by an intricate interplay between neural elements organized at microscale such as those mediating cellular plasticity and neurotransmission (including but not limited to the cholinergic system), and the macroscale level of neural organization. The latter includes an upregulation of residual brain systems underpinning the impaired cognitive function, brain systems involved in learning, and brain regions able to flexibly adapt to increasing task demands in the face of cognitive challenge imposed by brain injury, the so called ‘multiple demand’ cortex.^4^

Future work will need to investigate how the hippocampal-fornix and cholinergic systems interact to support recovery of memory. Further questions remain: How do these systems support other commonly affected cognitive domains such as language and attention, or motor recovery? And what is the contribution of other neurotransmitter systems to cognitive recovery? After all, distributed brain networks supporting working memory, attention and learning, such as the multiple demand cortex, are innervated not only by ascending cholinergic neurons, but also by noradrenergic, dopaminergic and serotonergic systems that project widely to subcortical and cortical regions. How do these neurotransmitter systems interact at a macroscale to support recovery of specific cognitive functions? What is the dose-response relationship between neurotransmission and cognitive function? This study only begins to address these questions.

Cholinergic medications, such as donepezil, are used for symptomatic treatment of memory impairment in Alzheimer’s disease, and although there is some emerging evidence that they may help improve cognition in patients with vascular cognitive impairment,^5^ their use is not always beneficial. In a randomized trial of auditory training in patients with aphasia, cholinergic treatment worsened comprehension, but there was a trend towards better naming on drug than placebo,^6^ suggesting that the effect of cholinergic enhancement is task specific, and/or that the dose-response function is more complex. There is evidence that cognitive-enhancing drugs working on neurotransmitter systems can have opposing effects on different cognitive tasks, and their relationship to performance is likely to be explained by a parabolic inverted U-shaped function.^7^ Hence, if cholinergic stimulation was already ‘high’ in the auditory cortex of the aforementioned study, increasing it further with cholinergic medication might have tipped performance over the vertex of the parabola and resulted in impaired performance. The current study provides support for using brain imaging to indirectly assess the status of the cholinergic system, and perhaps gauge whether neuropharmacological enhancement of the cholinergic system may be helpful in an individual patient.

In recent years, there has been growing excitement about deriving *in vivo* patient-specific measures of neurotransmitter status and relating these to brain function, recovery potential or response to cognitive enhancers. For example, in a study of patients with traumatic brain injury, clinically available dopamine Transporter imaging (DaT SPECT) was used to stratify patients into those with low or normal dopamine transporter status. Only those patients with a hypodopaminergic state in the caudate nuclei showed dopaminergic cognitive enhancement with methylphenidate. This promises a clinically applicable method of stratifying patients with brain injury for allocation to dopaminergic therapies.^8^ Likewise, in a recent randomized controlled trial in patients with Parkinson’s disease, only those with noradrenergic deficiency showed improvements with noradrenergic therapy in a response inhibition task. Here noradrenergic integrity was assessed using ultra-high field 7 T MRI of the locus coeruleus using a neuromelanin-sensitive magnetization transfer sequence.^9^

Despite theoretical beneficial effects of serotonin on promoting neural plasticity and the initial enthusiasm about using serotonergic treatment (fluoxetine) to improve recovery after stroke, recent randomized controlled trials have failed to show an overall benefit of serotonergic treatment on stroke recovery.^10^ These trials adopted a generalized approach whereby patients received fluoxetine therapy irrespective of their serotonergic status. Perhaps a more personalized approach based on serotonergic status would have improved the success rate of such trials. The principles and techniques demonstrated by O’Sullivan and colleagues^1^ could potentially usher in a new era of using neuroimaging to derive personalized neurotransmitter fingerprints with which to stratify patients in trials of cognitive enhancement therapy in the future, and thereby improve the likelihood of successful outcomes.
