# Correction to: Cholinergic neurotransmitter system: a potential marker for post-stroke cognitive recovery

**DOI:** 10.1093/brain/awac251

**Published:** 2022-08-03

Fatemeh Geranmayeh. Cholinergic neurotransmitter system: a potential marker for post-stroke cognitive recovery, *Brain*. 2022;145(5): 1576–1578. https://doi.org/10.1093/brain/awac142

In the originally published version of this manuscript, the schematic Figure 1 resulted in some confusion. The figure and associated legend have now been updated.

